# “If I go down, if I crumble, then everybody does” — identity crisis and emotional strain in parents of children with rare and undiagnosed conditions: a qualitative study

**DOI:** 10.1186/s13023-026-04384-5

**Published:** 2026-06-09

**Authors:** Tara Maria Hoffmann, Bettina Friedrich, Celine Lewis

**Affiliations:** Population, Policy and Practice Department, UCL Great Ormond Street Institute of Child Health, London, UK

**Keywords:** Rare condition, Undiagnosed condition, Mental health, Emotional wellbeing, Parent caregivers, Uncertainty, Genomic sequencing, Psychological counselling, Psychosocial insights, Family dynamics

## Abstract

**Background:**

Caring for a child with a rare condition can significantly impact parents’ emotional health, yet research on the emotional impact is limited. This qualitative interview study sought to investigate the lived experiences of parents who are experiencing higher psychological burden. Participants were parents of undiagnosed children undergoing whole genome sequencing (WGS) through the Genomic Medicine Service (GMS) across multiple NHS sites in England. Parents were purposively sampled to select those scoring poorly on validated measures of anxiety, family impact and/or resilience (GAD-7, PedsQL family functioning, BRS). We interviewed 24 parents after testing but prior to the return of WGS results. Questions focused on understanding their lived experience, in particular the emotional impact of their child’s condition and how they coped day-to-day.

**Results:**

Semi-structured interviews were transcribed and analysed using reflexive thematic analysis, leading to the generation of the central organising concept: “The construction of a caregiver identity: Torn between being a ‘hero(ine)’ and being a parent.” Parents’ experiences as caregivers often involved a significant re-evaluation of their parental role, as they sought to provide love and support while also adjusting to changes in their envisioned family life. Societal expectations, along with the complex challenges of navigating the health and social care systems, contributed to emotional strain. In particular, mothers often adapted their personal identities to embody a ‘heroic’ caregiving role. The anxiety caused by the uncertainty surrounding their child’s condition was also found to affect parents’ mental health. Areas for psychological interventions and recommendations to support parents’ mental health are identified.

**Conclusions:**

Further research is needed to explore how the return of genomic sequencing results impacts parents’ emotional wellbeing, and whether and which psychological counselling modalities throughout the journey might reduce emotional distress.

**Clinical trial number:**

Not applicable.

**Supplementary Information:**

The online version contains supplementary material available at https://doi.org/10.1186/s13023-026-04384-5.

## Background

### Caring for a child with a rare and undiagnosed condition

Individuals affected by rare conditions often experience a diagnostic delay, with the average time to achieve an accurate diagnosis being approximately 4–5 years [1]. Recent research is beginning to elucidate the emotional journey for these parents, including the mental health impact. This has shown that for some parents it is associated with anxiety and depression [2, 3], lower quality of life [4] as well as worry regarding their child’s quality of life [5]. Despite the fact that some families may benefit from mental health support services, awareness of those amongst families is limited [6]. Additionally, parents have reported a lack of access to support groups [7], leading to a sense of isolation [8].

Barriers to accessing support services may be because of family members not identifying themselves as ‘carers’, or “invisibility” due to lack of a diagnosis, or because of informal care-giving by family members, highlighting why it is important to define the term “carer” [9, 10]. Parents may struggle to reconcile their role as a caregiver and at the same time a parent, resulting in ambivalence about their own identity, a phenomenon previously identified in the geriatric caregiving context [11]. Approximately 50% of parent carers have never been asked about their emotional wellbeing by health professionals [5], highlighting a lack of awareness of the mental health impact amongst those involved in their child’s care. 

### Whole genome sequencing: a path to answers or more questions?

Whole genome sequencing (WGS) can determine the complete DNA sequence (protein-coding and non-coding regions) of a genome. Launched by the NHS England in 2012, the 100,000 Genomes Project used WGS to generate genomic data that provided actionable insights for those with rare conditions and other diseases, influencing patient care and treatment decisions [12]. The project’s success led to the establishment of the Genomic Medicine Service (GMS), which now allows WGS to be offered as a clinical service by specialists other than clinical geneticists [13]. As over 70% of rare conditions have a genetic cause, this technology may facilitate an earlier diagnosis - diagnostic yields of 38.6% have been reported compared to 7.8% from whole exome sequencing or ‘usual care’ [14]. However, this will still leave a significant number of parents without a genetic cause for their child’s condition.

Research has shown that parents want a diagnosis for a range of reasons including having a better understanding of the underlying genetic cause of their child’s condition, to alleviate feelings of guilt and to connect with other parents of children with the same condition. Yet, even with diagnosis, parents can still experience anxiety and uncertainty about the future, often because the condition is so rare that very little information is available [6, 15, 16]. Even when a diagnosis is *not* found, parents are unlikely to regret their decision to undergo genetic testing, despite research showing they may still feeling anxious towards the future [16, 17]. These findings emphasize that the support parents need should not be based on WGS results alone, but rather their particular needs in that moment [17]. Irrespective of a diagnosis, parents often experience stress and anxiety.

### Rationale for this study

Whilst it is evident that caring for a child with a rare condition has a significant emotional impact on some parents, research in this area is relatively sparse, particularly when compared to research on other outcomes such as the clinical impact of genomic testing, changes to clinical management, and diagnostic yields for various clinical indications [18]. Moreover, while many studies have focused on the use of quantitative questionnaires to understand parents’ emotional wellbeing, a qualitative body of evidence looking at this topic is only now beginning to emerge, primarily focusing on parents’ experiences accessing health care services, as well as the emotional challenges of caregiving [19]. Understanding the lived experiences and root causes behind poor emotional wellbeing [5] is crucial, particularly during the period when the condition has not yet been diagnosed and when uncertainty is therefore heightened.

The primary aim of the study is to understand parents’ lived experience as caregivers with a particular focus on the mental health impact of caring for a child with a rare and undiagnosed condition. The secondary aims are to identify areas where improvements may be needed to enhance the overall support provided to families, and to provide recommendations for practice. 

## Methods

### Study design

This study is embedded in a mixed methods longitudinal patient-centred evaluation of the Genomic Medicine Service with collaborating NHS sites across England, details of which have been previously published [20].

A qualitative research methodology was used to gain an in-depth, rich account of the lived experiences of parents. Semi-structured interviews allow participants the freedom to articulate their perspectives in their own language [21], which is invaluable when dealing with a highly sensitive topic amongst a marginalised group.

### Data sampling

A subset of parents who had completed a survey within four weeks of consenting for WGS as part of this patient-centred evaluation of the Genomic Medicine Service [20] (*n* = 394) were purposively sampled based on their scores on three validated measures related to emotional and family wellbeing. These were:

- The Generalised Anxiety Disorder − 7 (GAD − 7) [22] which is a seven-item measure that assesses the frequency with which the responder has experienced symptoms of anxiety over the past two weeks;
- The Paediatric Quality of Life Questionnaire, Family Impact Module (PedsQL Family Impact Module) [23]; which assess family impact from a physical, emotional, social, cognitive, communication and worry perspective;
- The Brief Resilience Scale (BRS) [24]; which relates to the responders’ ability to cope or recover from stress.

We judged these three measures to provide a multi-dimensional view of parents experiencing higher psychological burden; high anxiety levels reflect significant psychological distress, while lower family impact scores suggest that the child’s condition substantially disrupts family life and parental functioning. Lower resilience indicates a reduced capacity to recover from or adapt to stress. Therefore, the majority of our parents had scored moderate to severe anxiety on the GAD-7, and/or lower scores on the PedsQL Family Impact Module indicating lower functioning and/or lower scores on the Brief Resilience Scale indicating lower resilience.

Further selection was based on ensuring diverse geographical and ethnic backgrounds of participants as well as socio-economic background because the time-consuming carer burden may lead to unemployment [25–27]. The ethnic categories were carefully selected based on the UK government’s official classification of ethnic groups (“White or White British”, “Asian or Asian British”, “Black or Black British”, “Mixed”, “Other ethnic group”) [25]. In addition, we aimed to include both mothers and fathers as whilst mothers are often reported to be the primary caregiver, making them more vulnerable to poor mental health compared to fathers [2, 3, 28], research about fathers’ emotional wellbeing is scarce. A final factor we considered was the child’s condition (e.g. neurological, metabolic) due to some conditions being more often associated with behavioural problems which strongly correlate with parents feeling stressed [29, 30]. Interviews were conducted with primary caregivers (one per parental dyad).

### Recruitment

Overall, 74 potential interviewees were invited to participate in the study by email or via text message (as indicated by themselves in the survey). After two weeks without response, parents were followed up. This resulted in 27 parents considering participation, 11 decliners and 36 non-responders. The final sample consisted of 24 of 27 parents being included in the study (63% recruitment rate among responders). The remaining three declined further participation due to unforeseen circumstances.

All 24 interviews were conducted via telephone or using virtual meeting software between 27th June 2023 to 28th of March 2024 (*n* = 7 were conducted by BF; *n* = 17 were conducted by TMH). Participants were offered a £10 voucher for their participation.

### Data collection

The initial idea and focus of the study were developed in consultation with a patient advisory team. This team contributed to the overall mixed-methods study by helping to shape the study aims and objectives, reviewing and providing feedback on data collection tools, and supporting the interpretation of findings. The advisory group included parents of undiagnosed children, clinicians, genetic counsellors, and researchers.

During early discussions, the team proposed initial ideas for the interview topic guide (see Supplementary Material – topic guide). Authors CL and BF then developed the first draft of the semi-structured interview guide, which was reviewed with the founder of Rareminds, a UK non-profit organisation specialising in psychological counselling for those affected by rare conditions [31] as well as a researcher from the organisation. This collaboration helped ensure that the questions were appropriate and sensitively worded. Additionally, author TMH received brief psychological training from a therapist at Rareminds specialising in rare disease counselling, to support the handling of sensitive topics during interviews. Based on this training, minor revisions were made to further improve the wording of the interview guide.

All interviews were either recorded using an encrypted audio device or digitally via online virtual software and lasted between 20 to 100 min, (mean = 55 min) and transcribed verbatim. According to best practice guidelines, interviews were conducted alongside data analysis, focusing on data quality to meet study objectives rather than data saturation [32].

### Data analysis

A reflexive thematic analysis [33–35] was undertaken as it is not bound to a single underlying theoretical framework. We used deductive and inductive codes mirroring a descriptive and interpretivist approach, meaning that the analysis does not aim to separate description from interpretation, but rather it makes the interpretative lens transparent and grounded in the data.

In reflexive thematic analysis, the Results section typically includes both descriptive summaries of patterns across the data and the authors’ interpretative insights about what these patterns mean in the context of the research question, supported by participants’ direct quotes as evidence. Our intention in including interpretative commentary is to make visible how we constructed the themes and to show how we derived our analytic insights from participants’ own words. Ultimately, reflexive thematic analysis was chosen because it aligns with our interpretivist stance, which views knowledge as co-constructed and meaning as shaped by context. This method enabled us to explore how parents make sense of their experiences, consistent with this study’s aims, while acknowledging the active role of the researchers in the analytic process.

A subset analysis for inter-coder reliability was not performed due to the nature of the paradigm applied [36]. The analysis was facilitated through the Nvivo14 software. Following an initial listening of all interviews and multiple readings of the transcripts for data familiarisation, all interviews were coded three times in total. During the second round of coding, an iterative process involving the creation of mind maps based on the codes was used. This process led to the development of the central organising concept focused on identity and emotional strain (presented here). A second central organising concept, focused on how parents cope with grief amidst the uncertainty surrounding their child’s condition was also identified, and is the focus of a separate paper [37]. At this point, these concepts were analyzed independently; however, they provide complementary insights into the phenomenon under study.

Furthermore, descriptive statistics were generated using SPSS mac 29.0.2.0. PedQL scores were calculated according to the protocol [23].

### Research paradigm and researcher reflexivity

This study can be situated within a post-positivist research paradigm [38], adopting a critical realist ontology and contextualist epistemology [39]. Parents’ experiences are seen as their individual lived truth, influenced by socio-economic and cultural factors and the nature of the child’s condition, as opposed to relying on there being one objective truth. In order to account for this individual truth, a contextualist positionality towards the data was adopted to facilitate the existence of certain concepts, while simultaneously giving rise to new streams of thought [39].

In line with recommendations [33], TMH was engaged in reflexive journaling and designed mind maps to enable others to follow her streams of thoughts. In reflexive thematic analysis, the researcher takes on an active role in knowledge production, meaning that their own positionality to the data provides both advantages and challenges. TMH acknowledged her lack of personal experience in caring for a child with a rare condition and her position as a female researcher. However, she drew on her limited clinical experience of communicating as a training physician with families who care for a child with a rare condition.

Following recommendations for researchers unfamiliar with the research area of interest [40] TMH sought regular guidance through fortnightly debriefings with the supervisor with expertise in the field of genetic and genomic medicine as a behavioural scientist (CL). During the early analysis phase TMH also received helpful input from a psychologist from Rareminds as well as the study advisory team (see below), to support the trustworthiness of data interpretation.

### Patient and public involvement and advisory team

The evaluation project has a dedicated advisory team which meets every six months with the aim to ensure that the research questions are relevant, and the interests of the population being studied are adequately considered. For this particular sub-study, the topic guide content was discussed by the advisory team and the founder of the charity Rareminds (see above). Early interpretations from the analysis along with Recommendation for Practice were also discussed with the advisory team as well as with the founder and a researcher from Rareminds (see Acknowledgements).

## Results

### Participants characteristics

Study participants consisted of 4 men and 20 women (*n* = 24), ranging in age from 21 to 54 years (mean = 38 years). The majority identified as white British (Table 1).

**Table 1:** Participants’ characteristics

	(*n* = 24)	%
**Gender**		
female (n,%)	20	83.3
male (n,%)	4	16.7
**Age**		
age in years (mean; range)	38;21 - 54	
**Socioeconomical Characteristics**		
*Income (n*,*%)*		
Below £10,000	2	8.3
£10,001 to £30,000	6	25.0
£30,001 to £50,000	4	16.7
£50,001 to £70,000	7	29.2
Over £70,001	3	12.5
Prefer not to say	2	8.3
*Ethnicity (n*,*%)*		
White or White British	17	70.8
Asian or Asian British	5	20.8
Other ethnic group	1	4.2
Prefer not to say	1	4.2
*Religion (n*,*%)*		
None	10	41.7
Christian/Catholic	8	33.3
Muslim	3	12.5
Hindu	1	4.2
Sikh	1	4.2
Prefer not to say	1	4.2

Participants mean anxiety score was “moderate” (12.8, range 0–21) and mean resilience was reported as “low” (2.7, range 1–4). Cognitive Functioning was the most impacted aspect of the PedsQL Family Impact Module, followed by Family Relationships and Communication (see Table 2).

**Table 2:** Participants’ psycho-social characteristics

	(*n* = 24)		%
**Psychological Questionnaire Results**			
*Anxiety*			
GAD-7 score for anxiety^*1^(mean, range)	12.8; 0 - 21		
GAD-7 score category for anxiety^*2^(n,%)			
low	2		8.3
mild	7		29.2
moderate	4		16.7
severe	11		45.8
*Resilience*			
Brief Resilience Scale score^*3^(mean, range)	2.7; 1 - 4		
Resilience scale category (n,%)			
low	11		45.8
normal	13		54.2
high	0		0
**PedQl TM Family Impact Module** ^*4^			
Total Score^*5^*(mean*,* range)*	43; 10 - 80		
Subscores^*6^*(mean*,* range)*			
Physical functioning (max. 30 points)	14.5; 0 - 23		
Emotional functioning (max. 25 points)	13.0; 0 - 20		
Social Functioning (max. 20 points)	8.5; 0 - 15		
Cognitive Functioning (max. 25 points)	9.4; 0 - 20		
Communication (max. 15 points)	6.6; 0 - 12		
Worry^*3^ (max. 25 points)	12.6; 0 - 20		
Daily activities (max. 15 points)	7.5; 0 - 12		
Family relationships (max. 25 points)	8.9; 0 - 18		

^*1^Generalized Anxiety Disease Assessment based on 7 items from 0-21 with higher scores indicating higher anxiety

^*2^Anxiety categorised as: 0 to 4 “low”; 5 to 9 “mild”; 10 to 14 “moderate”; 15 to 21 “severe”

^*3^Brief resilience score categorised as : 1 to 2.99 “low”; 3 to 4.3 “medium”, 4.31 to 5.00 “high”

^*4^Numbers based on *n* = 23 responses due to missing response

^*5^Family Impact Module Score based on 36 items encompassing 8 scales with a total score from 0-100 with higher scores indicating better family functioning

^*6^Subscores possible responses: 1 = “never”; 2 = “almost never”; 3 = “sometimes”; 4 = “often”; 5 = “almost always”; higher scores indicate greater impact

**Table 3:** Characteristics of participants’ children and their disease

		(*n* = 24)		%
Age in years (mean; range)		4; 1-15		
**Disease category of child’s condition** (n,%)				
developmental disorder		13		54.2
developmental disorder & neurological disorder		1		4.2
musculoskeletal		1		4.2
neurological disorder		5		20.8
neurological disorder & musculoskeletal		2		8.3
ophthalmological disorder		1		4.2
ophthalmological disorder & metabolic disorder		1		4.2
**Severity of the condition** ^*1^ *(mean)*				
My child’s condition is serious			3.7	
My child’s condition has major consequences on their life			4.1	

^*1^self-reported questions on the severity of the child's condition. Possible answers were: “strongly disagree” = 1; “disagree” = 2; “neither agree nor disagree” = 3; “agree” = 4; “strongly agree” = 5

In terms of the clinical indication of the participants’ children, developmental conditions were most commonly reported (see Table 3). Most participants agreed that the child’s condition had a serious impact on their lives (mean = 4.1; “agree to strongly agree”) and perceived their child’s condition as severe (mean = 3.7; “neither agree nor disagree to agree”) (see Table 3).

### Qualitative results

**Fig. 1:**
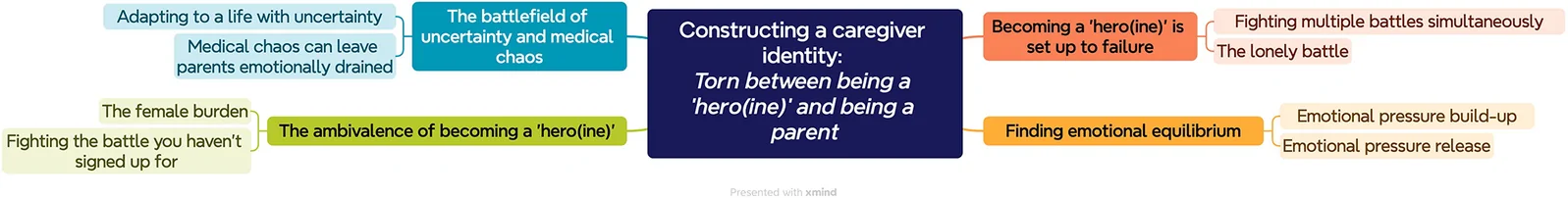
The central organising concept. This is a figurative summary of the central organising concept (center, dark blue), including themes (turquoise, green, orange, yellow) and related subthemes

The central organizing concept, “Constructing a caregiver identity: Torn between being a ‘hero(ine)’ and being a parent”, was shaped by four key underlying themes. Serving as an overarching interpretative concept, it encapsulates the lived experiences of caring for a child with a rare and undiagnosed condition, as outlined in the study’s first aim (see Fig. 1). All themes were supported by data from all interviews. Representative quotes from participants (names replaced by pseudonyms) have been used.

### The central organizing concept: “constructing a caregiver identity: torn between being a ‘hero(ine)’ and being a parent”

When parents find themselves in the unexpected role of being a caregiver for a child with a rare, undiagnosed condition, some undergo a significant reevaluation of their identity. At the same time as expressing their unconditional love for their child, they were also found to mourn the loss of their envisioned life for themselves and their family. This can cause an internal struggle which can make acceptance of the unanticipated caregiver role challenging, as it diverges from their pre-planned life trajectory. Caregivers, particularly mothers, immerse themselves in their new role, often reshaping or setting aside their prior identities, leading them to embody a ‘heroic’ archetype, which is associated with new expectations. Thus, parents find themselves amidst an identity crisis: torn between being a ‘hero(ine)’ and being a parent. The recurrent use of battle metaphors throughout participants’ accounts (e.g. “relentless”, “fight”, “battlefield”, “hero”, “crumble”) further underscores how caregiving was experienced as a prolonged and emotional struggle shaped by uncertainty, advocacy and emotional resilience. 

Their caregiver role introduces parents to novel tasks, which they look to accomplish, despite difficulties adapting to these new circumstances. Due to the uniqueness of their situation, parents often face societal judgment and can find it hard to maintain relationships, exacerbating their sense of isolation. Furthermore, the uncertainty surrounding rare conditions complicates future planning. This perpetual state, combined with the multifaceted responsibilities they are required to take on alongside an unresponsive health service, can generate significant emotional strain. Failure to manage these emotions can precipitate an imbalance detrimental to parents’ mental health.

### Theme 1: The battlefield of uncertainty and medical chaos

This theme describes the context and intervening conditions parents find themselves in. The beginning of parents’ journey may have come as a shock: *“So when they told us I was shocked because…I didn’t even know that I had to be worried about it”* [Anjali]; or parents realized more gradually *“…that something was not right”* [Mary]. Both scenarios share the element of unforeseen disruption, leaving parents unprepared in the face of health system and personal challenges around their child’s condition.

#### Subtheme 1: Adapting to a life with uncertainty

Subtheme 1 dives into the emotional impact on families, which leads to some parents feeling *“like your future’s been taken away…”* [Ingrid]. Due to the lack of a diagnosis, parents frequently spoke of fearing the worst but remaining hopeful for the best: *”…suddenly you’re faced with the very real possibility that your child might pass away…and all the time you’re hoping for the best but fearing the worst.”* [Ingrid]. Some seek control over their child’s situation, trying to make the best out of uncertainty:

*I need to understand what I’m dealing with and there wasn’t a lot of that…I do worry about what’s going to happen to [child’s name] …but nobody has told us this is going to happen …so I just feel a bit lost…* [Emily]

This feeling of being *“lost”*, was exacerbated by parents feeling that knowledge was not being shared by medical professionals. Uncertainty around when parents would receive diagnostic test results was commonly expressed, which led to parents feeling helpless because of the lack of knowledge about the condition.

Participants appeared to have mixed feelings about getting a diagnosis. Fearful of being presented with a “fait accompli” diagnosis, some hope “*that it doesn’t show anything*” [Eric]. For other, living in an uncertain state had its benefits: “*it’s like burying your head in the sand”* [Lisa]. While uncertainty may evoke feelings of anxiety, it can provide a sense of comfort. Some parents subscribe to the notion that *“ignorance is bliss*” [Ingrid]. This intrinsic defense mechanism helps parents remain optimistic and protects them from the potentially upsetting prognosis of a definitive diagnosis. This may be seen as an adaptive response to the stimulus of uncertainty. However, such resilience appears fragile in the face of adversity, as demonstrated by Samipa, who stresses the emotional burden that uncertainty places on parents:

*I just tell myself [my child will] be fine and … [my child is] going to have a good quality of life*,* but the truth and the reality when you think about it*,* it really brings you down… I cry a lot some days*

Furthermore, Ingrid reflects on her statement “*ignorance is bliss*”: “…*the days following when you’ve had contact with the hospital and maybe hadn’t had the news that you were hoping… those days are particularly hard.*” This sentiment, that appointments can be particularly difficult emotionally, was frequently expressed by parents. Harroop describes how her resilience is tested when she has a clinical appointment coming up, and that this leads to anxiety because of the worry that something has been found that she was not hoping for:

*it still stresses me out like when we have an appointment…the week before I will be stressed out*,* …because I’m thinking ‘have they found something…is there something bad’*,* there’s always that anxiety whenever an appointment’s coming up just because I don’t know*

#### Subtheme 2: Medical chaos can leave parents emotionally drained

Parents frequently encounter barriers to receiving medical support. This subtheme describes the “medical chaos”, which leads these parents to feel they are in a “relentless fight”, leaving them emotionally drained, as described by Laura: *“…I’m usually the one that will take people to task if needs be – but sometimes I’ve just run out of steam completely.”* The use of the term *“usually”* indicates that she not only perceives herself as the person that needs to fight for the best medical care for her child, but also suggests that needing to overcom obstacles is not uncomon for her. However, as she describes, sometimes it becomes too overwhelming, and she runs *“out of steam”.*

Parents had various experiences related to the quality of care and support they received. Laura is grateful for the care received from the medical team in a tertiary care setting who acknowledge her as an expert in her child’s care, commenting *“that staff are much more willing to listen to me and trust what I’m saying”.* However, she has had *“bad experiences”* due to the *“much lower quality”* of care in the primary care setting, where she wasn’t taken seriously by medical professionals. She also feels that there is a lack of understanding among medical professionals about what it is like for her being a carer for her child:

*many therapists say to me ‘oh we won’t do the therapy with [child]*,* we’ll teach you how to do it and then you can build it into your everyday life’ – that’s just asking an awful lot of someone who has been thrust into this journey without any warning*

In this case, Laura felt put under pressure by the medical team to take on additional care roles.

This discrepancy in the quality of care was reiterated throughout interviews. Victor’s experience expands our understanding. The leading symptoms of his child’s rare and undiagnosed condition were assigned to a different specialty (cancer), resulting in his child’s referral and reclassification within the hospital organization. He describes how, unlike with more established care pathways such as cancer, children with rare conditions don’t have an established care pathway to guide them:

*there’s a real contrast between the support … and help afforded to families who are dealing with cancer*,* complete contrast with the stuff that is out there for kids with rare genetic conditions.*.*obviously your kid having cancer is the last thing that anyone wants to hear but*,*…it kind of unlocked this door*

### Theme 2: The ambivalence of becoming a ‘hero(ine)’

The second theme describes an expectation that these parents are seen, either by themselves or others, as ‘hero(ine)s’, depicting parents’ achievements as superhuman. For Laura, by distinguishing herself from others by saying: *“…my fellow medical mums and me*,* endearingly call everyone else “muggles” from Harry Potter!”*, she internalizes the ‘heroic’ role as a form of recognition. Reconceptualizing one’s identity as a ‘hero(ine)’ may be seen as a way to take charge or recover a sense of identity or purpose, but at the same time the need to be strong and fight for their child can be an exhausting emotional and physical burden, often carried by mothers.

#### Subtheme 1: The female burden

Subtheme 1 looks at the experience of mothers, like Susan, many of whom carry the responsibility of being the primary carer, often having given up their careers in order to become full-time carers: *“I had a 21 year career and I’ve stopped that…*,* I will no longer be working at all because I just cannot cope with the demands on me.”* She puts emphasis on the significant role that her career played in shaping her identity, which she now feels has had to end because of her new role as the primary caregiver. Substituting their job with the perhaps ‘invisible’ job of caregiving adds to mothers feeling pressured to succeed in that role, as described by Laura: *“…especially as a woman [it] is ingrained into you*,* …I felt a deep self-inflicted pressure that OK my husband’s at work all day*,* because I do work as well but it’s not paid*,* because I’m caring for my son*,* he’s at work all day so I must achieve everything…”*.

Interestingly, Laura’s husband’s willingness to share the caregiving responsibilities demonstrates an awareness of the ‘female burden’: *“…my husband kept saying to me ‘why are you all of a sudden do you think you’ve got responsibility for everything – you don’t.’.”*.

In contrast, Ralph’s recognition of his wife’s dedication highlights the asymmetry in their contributions to their child’s care: *“…she’s doing I would say at least 90% of the hard work…I’m only doing about 10% of the work that she is doing and even though that 10% I feel so tired so I can only imagine how she feels…”*.

This incongruency around caregiving responsibilities between both partners is also evident in the dynamic evolution of Anjali’s relationship with her husband. Her initial expectation of a shared experience transitions to resentment when she perceives her husband’s struggles as self-centered:

…*at first it was like…we’re both going through the same thing*,* but then when he was finding it very difficult*,* I became quite resentful towards him*,* because I couldn’t understand how he was making it about himself – so I thought he was being selfish…I didn’t feel that I could relate to him*

Her comment resonates with the archetype of the self-sacrificing mother who is inclined to put her own needs aside for the sake of her child, which is why, perhaps, she was unable to empathise with her husband’s emotional situation. This hints at an imbalance in the emotional burden associated with caring for a child with a rare condition, with potential implications for family and relationship dynamics.

Harroop, who is a single mother and the main caregiver, also expresses her dissatisfaction related to the inbalance of care-giving responsibility not equally shared with her ex-partner: *“We’re separated but I expressed a lot of the time to him that I find it really unfair that I’m taking on so much responsibility and mental load.”;*

Some mothers expressed envy that their partners were able to continue to work unlike they were: *“when… we both were working*,* it was almost arguing about who would be the one to care for [child] …you feel torn…between everything*,* which is why I gave up work*,* but then…I probably resent that…”* [Ingrid] Her comment implies a sense of resignation to societal expectations placed on women.

#### Subtheme 2: Fighting the battle you haven’t signed up for

Subtheme 2 looks deeper into the complex mix of emotions underlying parents’ emotional connection to their child’s well-being, and at the same time their reluctance to taking on the role of a ‘hero(ine)’ which is bound to sacrifice. The “battle you haven’t signed up for” is not only the caregiving role itself; it also suggests a battle against thoughts and feelings that clash with societal expectations of what a loving parent should feel.

Laura admires the resilience of single mothers going through the ‘heroic’ journey of caring for a child with a rare condition.

…*single mums doing this journey on their own…I just think they’re heroes because - I don’t like saying I don’t know how they do it because when people say that to me*,* I feel like saying I’ve got no choice –…but I certainly wouldn’t want to…*

Her account illustrates the tension between the social valorisation of caregiving and the lived experience of responsibility. While she admires single mothers and describes them as “heroes”, she simultaneously recognises that, much like herself, they often continue because they feel they have no alternative. Her reflection highlights the emotional complexity of a role that is both deeply meaningful and experienced as an unavoidable obligation.

Susan admits that there have been moments when the emotional strain has become too much and she has felt tempted to abandon her caregiving role: “…*there have been points when I’ve just wanted to walk out. And I’ve literally been like I could just walk out now and not come back.*” Similarly, Harroop acknowledges at times feeling resentful of having to take on caregiving duties alone.

…*I don’t rely on anyone*,* …it’s just…that I’ve gotten used to it*,* but I just feel annoyed…that I’m in a situation where I have to do it on my own because there is no other choice*

The perception among parents that they have “no choice” but to be the main caregiver were sometimes challenged by intrusive thoughts which do not conform with society’s expectations on motherhood, nor fit with the ‘heroic’ persona. These thoughts, however, could be particularly distressing and stigmatizing, as illustrated by Laura’s internal conflict:

…*it can feel very scary to say some of the things you feel out loud about feeling resentful towards your child…intrusive thoughts are a scary thing to talk about*

Laura’s confession evokes her fear at being judged a ‘bad mother’ as well as the limited space she feels she has to express negative emotions. Anna further highlights the challenging internal thoughts she has had at times, especially when faced with other people's comments about her situation.

…*I hate it when people say*,* when you’ve got a child with special needs*,* that there’s a grieving process because I think that’s not fair on the child to say things like that*,* because I wouldn’t not have [my child]*,* but it would make the world easier for her and for us a little bit…*

Yet parents were also keen to express the unconditional love they had for their child. Maya comments: *“…Because as far as I’m concerned*,* as long as he’s happy*,* that’s all that matters to me.”* [Maya]. This statement highlights how she gains personal fulfillment from her child's happiness Anjali offers further insight into this dynamic, expressing how her child’s enduring happiness and love served as catalysts for a shift in her perspective:

*[child] …has made us change our way of thinking because [child]’s just always so happy and…loves us both to pieces and what more could you ask for…so I think…let’s enjoy our lives together*

Hence, for certain parents, their commitment in caring for their child provides a profound sense of purpose, yet at the same time it is intertwined with more complex, difficult emotions including at times, anger and resentment towards the caregiving role and the situation they find themselves in.

### Theme 3: Becoming a ‘hero(ine)’ is set up to failure

The third theme highlights how becoming the ‘hero(ine)’, which is inherently subject to many trials and hardships, can lead to parents feeling pressured to take on more than they would otherwise be expected, as Susan explains: *“Well if I go down*,* if I crumble*,* then everybody does*,* the whole family will so I have to [cope].”* The emotional pressure of being the primary caregiver can also result in parents experiencing isolation, as they refrain from sharing their thoughts and emotions with others. Mala articulates this sentiment: *“I didn’t want to put everybody else into that worrying frame of mind*,* so I would…just keep it to myself.”*

#### Subtheme 1: Fighting multiple battles simultaneously

This subtheme describes the many concurrent roles taken on by parents, as articulated by Valentina who describes having to perform almost super-human acts: *“I tried to pretty much multiply myself to try and be present for everybody…”*, Yet, the demand for these 'heroic', superhuman abilities is doomed to failure. Anna provides insights into her feelings of self-doubt and guilt regarding her poor work performance:

*I was having to drive [my child]*,*…and try and get my work done*,* because I can’t do it in the evenings because of how [my child] is*,* … not pulling my weight at all really at work…I knew that I wasn’t doing my job very well*

Daily organisation plays a major role in meeting the demands parents face so that they can fit in the many tasks required in both their work life and parenting life. Organisation is also key to ensuring the child’s medical safety. For instance, Catlyn adopts a proactive approach to ensuring her child's safety at nursery by implementing certain measures:

…*So the nursery have got that [an action plan in case of seizures]. I also showed them a video of what [child’s name]’s seizures look like because they’re not typical seizures so you might not be aware that it’s happening. …if [child’s name]’s even slightly poorly…[child] stays at home with me*

Catlyn demonstrates her expertise in navigating her child’s condition, drawing upon her personal experiences as she explains how the nursery team can know when her child looks *“poorly”*.

Parents sometimes feel they have to becoming overly protective as a result of their child’s condition. Harroop gives the explanation for her protective behaviour as being driven by the uncertainty around the condition: *“If anything*,* I feel like I’m more overprotective of [my child] because I have no idea what’s going on*…”. Ralph also decribes the need for constant supervision of his child to prevent any potential risks:

…you have to supervise [child] all the time, you can’t let your guard down,…You need to be aware all the time to try and avoid certain situations because once they happen then it’s too late

Ralph’s acknowledgment that any lapse in supervision can result in immediate and irreversible consequences, illustrates the heightened level of responsibility and stress these parents endure, resulting in a ’hyper-vigilant’ state of mind.

#### Subtheme 2: The lonely battle

Subtheme 2 elucidates the additional factors contributing to the perceived isolation frequently reported by parents. For Emily, a sense of isolation stems from a perceived lack of understanding (*“…people just don’t understand…”)*, which she finds frustrating, thus she tries to *“raise awareness”* of her child’s condition to combat societal ignorance. This sense of isolation is further compounded when parents attempt to seek support but encounter barriers. Ingrid’s account of reaching the limits of her ability to cope and the emotional difficulty of asking for help illustrates the complex interplay between individual vulnerability and external pressures faced by parents:

…*it took so much to actually ask for help…you feel lack of pride*,* you feel worthless and then to be told ‘well actually you don’t need help’*,* I thought well I wouldn’t be asking for help if I didn’t need it…*

For Ingrid, seeking help entailed stepping out of her ‘heroic’ caregiving role, which she associates with a diminished self-worth and a loss of independence – both central to her sense of identity.

In contrast, some parents adopt a position of strong self-reliance, which may also contribute to isolation in different ways. Anjali’s assertion that she and her partner *“obviously…know [our child] the best”* implies a strong sense of confidence in her and her partner’s caregiving abilities. In stating that: *“…we know how to look after [child] – so I don’t rely on a lot of people for help.”*, Anjali places her and her partner as the primary experts in their child’s care. However, this self-reliant stance carries an emotional cost, as she later acknowledges: *“I guess [it] takes a toll on my mental health too because I don’t really have that much time to myself”.*

Stigma and perceived societal attitudes toward disability further intensify parents’ isolation. Eric shares his experience with comparisons by other people he found hurtful:

…*my cousin… had a baby born… prior than Emma but …normal …And there was a lot of comparisons*,*…comments along the lines of “.Look at the size difference”… And I don’t think there was any malice to it but that really upset me. …it makes you think well*,* you don’t want to see these people because you don’t want to expose yourself to those sorts of comments*

These comparisons reinforced his sense of being “different”, prompting withdrawal from social interactions, albeit at the cost of increased isolation. Some parents also reported feeling shunned by friends and family who stopped socializing with them or inviting them to events, further contributing to a sense of having to nativate the caregiving journey alone. For instance, Ingrid shared: *“people are fed up of hearing things about [the condition] and I get it…some people don’t know how to cope with disability…some people…have definitely backed off*,* you’re not invited to things sometimes or they won’t come to things that you arrange….” *

Moreover, some parents faced system-wide challenges as a result of their child’s condition, Anna, for instance, reported difficulties with her child’s school, which she perceived as lacking inclusivity: *“…they’d made a decision already - and they weren’t trying their best for [my child].”* This account suggests a perceived institutional failure, contributing to feelings of betrayal and anger. Her subsequent expressions of feeling “lost, struggling, [and] alone” further underscore the emotional isolation associated with this experience. Similarly, Barbara’s concern about societal stigma, illustrated by her observation of people who *“stare at [her child]…and shake their heads.”* suggests an anxiety linked to public scrutiny.

### Theme 4: Finding emotional equilibrium

As exemplified in theme 3, taking on the role of ‘hero(ine)’ can place considerable strain on parents’ emotional well-being, as they push themselves to their own mental and physical limits. Being the hero(ine) can be seen as a psychological defense mechanism against more vulnerable, difficult and complex feelings such as helplessness, anger, and guilt that result from being subject to forces outside one’s control. This can result in parents denying their own needs around care and comfort, which can, in some cases, lead to destructive behaviours in order to have those needs met.

#### Subtheme 1: Emotional pressure build-up

Subtheme 1 looks deeper into the roots of the emotional distress that may be caused by obstacles attributed to the “heroic” role. Jessica’s comment *“…And they [the child] dominate every moment of your life…”* suggests that the intense demands placed on her permeate every aspect of her life, leaving little room for personal time. Within this context, some parents maintain a façade of emotional stability to remain “functional”. As Emily reflects: *“I realise that…it’s a lot harder to digest than what I try and have to portray. So I try to pretend I’m doing OK.”* Emily’s account of *“pretend[ing]”* highlights the pressure to appear resilient despite underlying difficulties, accentuating the tension between genuine emotions and the socially expected image of the ‘heroic’ caregiver. This cognitive dissonance between outward presentation and internal reality can exacerbate parents’ emotional strain.

Moreover, the expectation that parents should develop ‘expertise*’* in their child's care adds to a feeling of pressure and exposes them to feelings of vulnerability, as illustrated by Mary’s experience of feeling both judged and unsupported during her interactions with medical professionals:

… *then the nurse turns round and almost makes you feel like you’ve done wrong when actually I’ve never been in this world doing chemo on my child. So I need your support in the hospital to help me get through this*

This pressure may drive parents, as is the case with Samipa, to immerse themselves in research and information-seeking behaviors: *“I go and look up on Google what is the medicine or is there going to be a cure*,* are they doing any research in the future*,* is there going to be any cure for this condition – I keep on reading up.*

Moreover, the self-imposed act of pushing oneself to mental and physical limits is in itself an emotional burden. For instance, although Harroop admits that she needs a break, she pushes herself to function like a “robot” by neglecting her needs:

*I just want to be alone*,* but obviously because I have children I can’t be alone…it’s like I’m in that mode of it’s just robotic*,* …I’m just doing the daily things of what I know I have to be doing*

Harroop’s comment suggests she has found a way to emotionally disengage from many of her daily tasks. This super-human existence ultimately results in a profound sense of losing oneself, which resonates with Jessica’s statement that caring for her child *“takes its toll”*.

Ralph reflects on the loss of his former identity, describing a dramatic shift from having an active, independent lifestyle to being in a state of perpetual exhaustion:

*I was good*,* I was doing things on my own*,* …a lot of that has changed*,* I had lots of energy*,* it’s just the stuff all disappeared now which*,* like I said*,*.I wake up feeling tired*,* I go to sleep feeling tired*

Talking about his identity in both the past and present tense suggests a perceived rupture in his identity. He conveys a sense of involuntary loss, suggesting that this transformation has been imposed rather than chosen.

In addition, some parents spoke about the burden of feeling guilty due to not being able to give as much attention to other children, leaving them emotionally torn. Valentina highlights how her obligation to spend much of her time in hospital enhances her sense of guilt about her other child:

*Obviously guilt was already a feeling that you have as a mother of a second child… particularly if you are breastfeeding because you have to spend 90% of the time with the new one*,* so that’s like a standard feeling for a mother*,* imagine if you have to be away for twenty days [in the hospital] and also pay extra attention…*

#### Subtheme 2: Emotional pressure release

To alleviate the pressure of daily life including frequently denying their own care needs and wellbeing, parents were found to develop coping mechanisms. For instance, some parents engage in short-term comfort behaviors that are ultimately destructive, such as *“drinking a bit too heavily”* [Victor] or *“go[ing] to the kitchen cupboard if things are overwhelming”* [Jessica]. Harroop acknowledges that she would cope with her emotional distress around the time of her child’s appointments by going on shopping sprees:

…*I would go to the shopping centre and start spending money in order to get [emotional release]…I…didn’t know how to process…every time [child] had an appointment I was spending beyond my means…*

Recognizing these behaviours as destructive, some parents shift to adopting healthier long-term strategies for emotional release, such as cognitive disengagement and physical exercise, as highlighted by Emma and Thomas:

…*sometimes just having a break away from [my child] and not thinking about it does my mental health and my anxiety that little bit of good.”* [Emma]

*We have to…make some time for ourselves and…get some exercise and get out…into the garden to try and stay positive.* [Thomas]

Some parents recognize the importance of nurturing their own needs, thus they remodel their attitudes towards their caregiving role. Samipa describes her thoughts related to this intricate change of mind set:

…*because if we are good and healthy…I can be there for my [child]*,* so…I need to…[look] after my health a bit more and be strong mentally and physically for [my child]…for this family as a whole…*

She suggests that regaining a balance between self-care and the demands of caring for a child with complex needs is important to her, emphasizing that she now prioritizes her own needs more.

Similarly, Laura describes how she used to set high expectations for herself, as evidenced by her daily routine: *“…I must make sure the house is spick and span*,* do all the washing*,* get all my son’s medical admin done…”.* Letting go of this mindset has been challenging for her because it induces a sense of guilt:

… *So that’s been one of the biggest challenges*,* is not feeling guilt for resting and for not having the whole house under control all the time*,* that’s been a huge shift for me…*

The “*huge shift*” in her mindset also includes asking others for support which she recognises as being vital to her own wellbeing: “*…And I guess that’s at the root of all of it, is asking people for what I need is the most important thing to my mental health.*” [Laura].

Some parents were also found to cognitively restructure the way they thought about their situation as a way of coping. For instance, Nilay reflects on her child’s condition by comparing it to that of other children with, from her point of view, more severe challenges and in this way reframes her situation through a lens of gratitude:

*I’m just like we are so grateful to be here [England]*,* to be able to get those things [therapies] because I know maybe in different countries or whatever they don’t do such things and we are very grateful that … [child]’s able to progress*

This cognitive reframing also involves making an introspective evaluation of one’s emotional state: “…*if I feel myself getting upset and uptight then I have to stop and just think OK I just need to go about this a totally different way.*” [Anna]. In some cases, parents sought psychological support externally: “*…[the mental health team] just help[s] me in regards to my emotions…*” [Louise].

Moreover, support groups were found to provide the comfort of a safe space to share experiences, combating the social withdrawal that threatens emotional wellbeing. Emma describes this as an opportunity to gain mutual understanding:

…*I think it’s more relating to the situation and that they understand the stresses that it can bring to you as a parent. They can empathise with you and they’re walking in the same shoes as you…*

Social networks and supportive work colleagues were also important, as emphasised by Nilay who comments: “*…but having someone like family or a good friend with you at all times that you can talk to about your feelings has helped me a lot…*”.

In contrast, Catlyn left her online support groups after finding the discussions “quite distressing”. She felt exposed to a lot of negativity, which she sought to avoid: *“…a lot of people talking about the worst case scenarios that happened with their children. I thought it’s not helpful…”*. By reducing her exposure to these anxiety-provoking stimuli, she manages her anxiety more effectively. Laura also experienced frustration from feeling like she and her child did not really fit in any support groups due to the uniqueness of her child’s condition:

*I don’t attend support groups for specific conditions because there are so many comorbidities that it just doesn’t help—I find it frustrating instead*

### Recommendations for practice

A second aim of the study was to develop a set of recommendations for practice, which relate to obstacles parents most frequently experienced along their journey (see Table 4).

**Table 4:** Identified recommendations for practice

Recommendation	Justification	Associated quote (examples)
**Recommendation 1**: Offer counselling to parents at different time points in their journey.	Parents found themselves in difficult emotional states at various times throughout their journey, which differs due to individual experiences.	*“…I think maybe having an opportunity*,* like an appointment for counselling actually in tandem with genetic results*,* because I think you need to talk to someone about it…”* [Ingrid] *“I really feel like they should have… some kind of trained therapist… present in the maternity units of hospitals*,*… when you’ve just had a baby you’re feeling vulnerable anyway but especially when you have a baby who’s got this undiagnosed condition…”* [Mala]
**Recommendation 2**: Identify and tailor support, advice, and information to the requirements of the particular family.	Some parents felt that their healthcare team did not provide the information, support, and advice that they would have liked.	*“I think … there should be…support…advice and information given out*,* because I never had it from the hospital…”*.[Emma]
**Recommendation 3**: Facilitate streamlined and shared access to information among healthcare providers.	Parents expressed a strong desire for a standardized system to simplify appointment scheduling and facilitate the sharing of their child’s health data among all involved healthcare providers.	*“I thought why am I having to coordinate all of this? Surely there’s got to be a better system…”* [Ingrid]
**Recommendation 4**: Assign a dedicated contact person to support and guide parents.	Parents often reported feeling isolated in managing their daily challenges, necessitating self-advocacy. Consequently, parents recommended the availability of advocates to provide assistance.	*“…I get frustrated because I feel like we’re in limbo all the time…And then he gets appointments and they’re cancelled and I just feel like there’s no one in contact with us…”* [Maya]
**Recommendation 5**: Maintain regular communication with parents to address questions and uncertainties associated with rare conditions because small, consistent interventions can provide significant reassurance.	Anxiety stemming from uncertainty greatly impacts parents’ mental health and should be addressed as far as possible by healthcare professionals. The dynamic nature of rare conditions often leaves parents fearing they will be “forgotten” due to insufficient feedback from medical professionals.	*“You’d be more effective to check up regularly… because my questions changed over time*,* so when we first spoke to [doctor] we had a different situation than now…”* [Valentina]
**Recommendation 6**: Increase sensibility among health care professionals when working with families affected by complex conditions. This includes their sensitivity to the emotional pressures that can be experienced by parent caregivers including grief, guilt, chronic uncertainty, and emotional overload, which may be hidden under an outward façade of resilience.	It is crucial that clinicians are equipped with the ability to effectively communicate and collaborate with the parents and to understand related emotional factors.	*“… better training for all healthcare * *professionals on understanding what parent carers go through and how their actions impact carers and how they are torn and their emotional availability and the way that they relay information and understanding the pressure that they’re under…”* [Laura]

These recommendations were identified through our analysis and reflect the societal and organisational challenges described by parents. They address key areas such as disjointed care pathways, lack of emotional and psychological support, poor communication from professionals, and the burden of navigating complex systems. By highlighting these systemic issues, the recommendations aim to inform more responsive, coordinated, and compassionate care practices that recognise and address the broader structural barriers impacting families. It is important to situate parental distress, as highlighted throughout the central organising theme, within a wider systems context. Parents accounts and recommenations highlight gaps, such as insufficient support services alongside prevailing societal attitudes that exclude or marginalize those families coping with disabilities. Labels such as ‘hero’ may reinforce expectations of resilience and self-sacrifice. This in turn may undermine recognition of families' support needs and hinder the implementation of much needed support services.

## Discussion

### Summary

The central organising concept “Constructing a caregiver identity: Torn between being a ‘hero(ine)’ and being a parent” encompasses parents’ reconceptualization of their role in the context of caring for a child with a rare and undiagnosed disease. Becoming the ‘hero(ine)’ enables parents to recover a sense of meaning borne from a perceived identify crisis around what it means to be a parent and at the same time a caregiver. At the same time, it also confers ‘moral value’ both internally in how one sees oneself, as well as externally in the ‘eyes of the world’. Feeling consumed at times by the challenges of caregiving, parents are placed in a stressful, yet vulnerable state. Rather than giving in to intrusive or negative thoughts, we propose that parents, and mothers in particular, find it helpful to adopt the ‘heroic’ persona as a psychological defense mechanism against societal expectations and the challenges they face. This study also highlights the negative impact of anxiety related to uncertainty and isolation on parents’ mental health. The interviewed parents offered novel perspectives on family and relationship dynamics, providing intriguing psychological insights at a unique timepoint during the ‘diagnostic odyssey’, as parents await WGS results for their child.

### Application to mental health research and theory

Parents finding themselves in the role of a caregiver report a spectrum of emotions as they navigate the ambiguous process of redefining their identity. Many of our findings resonate with those highlighted in a recent review [19]. These include: shock [41], which evolves into pervasive anxiety for the entire family due to uncertainties surrounding their child’s condition [42], feelings of frustration as they struggle with the loss of anticipated parenthood [43], renewed hope while awaiting results from genomic testing [17], and a desire for information about their child’s condition [15]. Interviewed parents experienced emotional exhaustion as a result of the intensity of caring for their child, which in turn could undermine confidence in their parenting abilities. This is contrasted by the need to “portray” emotional stability, or even a ‘heroic’ role, as exemplified in the ‘super-human’ narrative explored in the analysis.

**Fig. 2:**
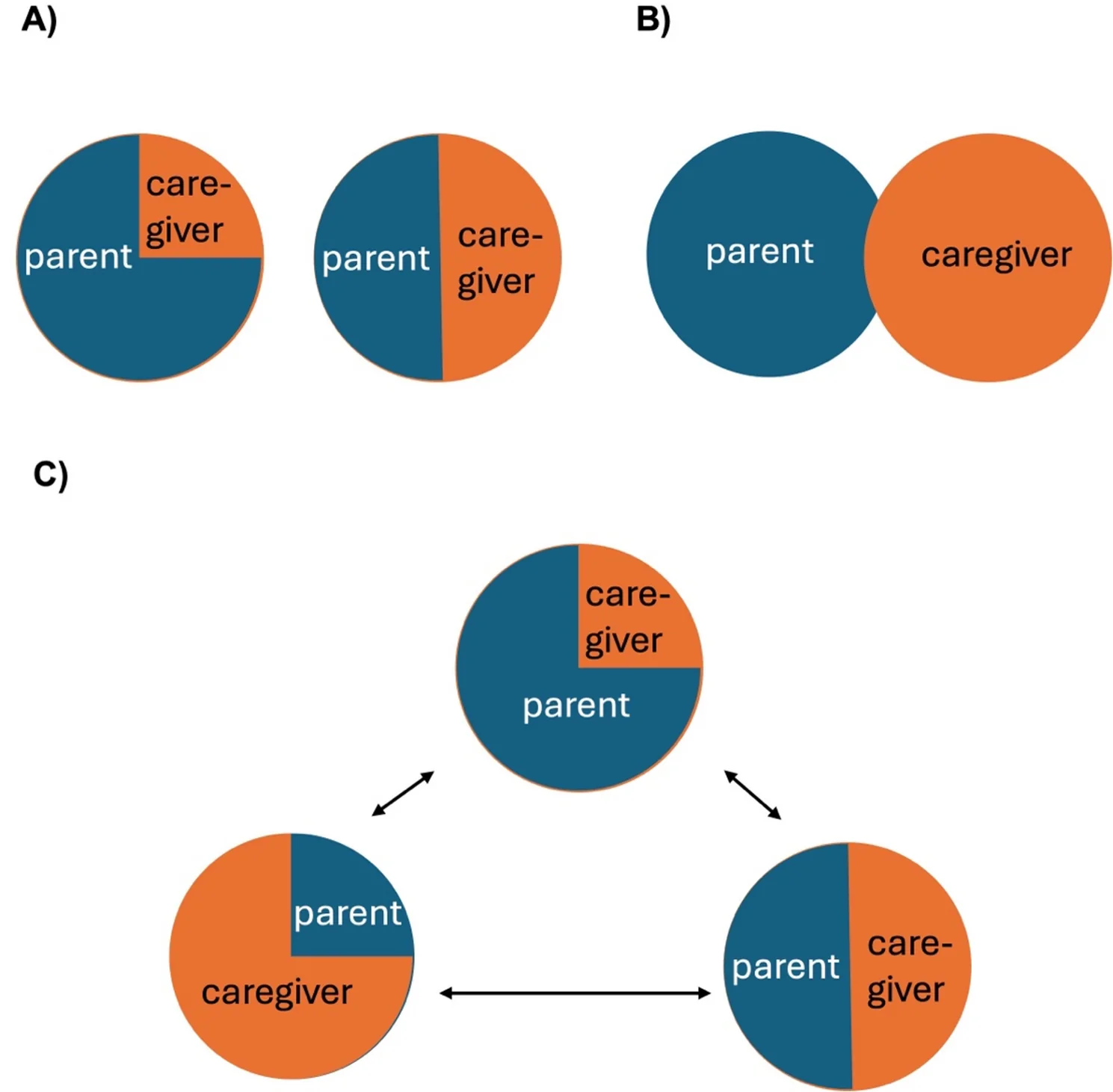
A proposed model for developing a parental caregiving identity in a rare disease context adapted from Montgomery and Kosloski, 2013 [53], with kind permission by Montgomery R. (**A**) The intensity of the identity crisis varies according to the child’s unpredictable condition; (**B**) High expectations are set by both parents and society for caregiving with parents assuming the dual roles of both parent and caregiver; (**C**) Parenting and caregiving identities evolve dynamically over time, shaped by the child’s changing condition and the shifting emotional and practical demands placed on parents

Parent caregivers are increasingly depicted as ‘heroes’ in both media and research to make their efforts ‘visible’ [44–47]. This portrayal may motivate parents to embrace the ‘heroic’ role for recognition, but it can also exacerbate their sense of isolation as society places more responsibility on them, as recently discussed in a report by Care Alliance Ireland [48]. Our findings shed new light on this scarcely investigated phenomenon among parent caregivers. The ‘heroic’ role places the normative task of parenting into a ‘super-human’ context due to the unique challenges of caring for a child with a rare disease. This often leads to parents feeling, or even desiring, isolation, as expressed in this study. The feeling of isolation may be particularly heightened in mothers who experience a disrupted narrative of ‘typical’ motherhood, e.g. due to the behavioural differences of neurodiverse children [49]. Referring to the “The Caregiver Model” by Montgomery et al. [50], initially developed for geriatric family caregiving, caregiving is seen as a dynamic process. It progresses from existing familial roles through several phases, culminating in an almost full caregiver identity, until they receive external support which reduces the workload, and the original identity becomes more prominent again. In parallel with our findings, loss of identity due to *“engulfment”* in the caregiver role was reported to be more common among female caregivers in the geriatric setting [51]. However, our proposal differs in that parents in this cohort may never achieve “full” transition (see Fig. 2). Consequently, psychological counselling should aim to assist parents in adapting to this ongoing emotional state. The significant distinction from Montgomery’s model is articulated in Theme 4. Parents perceive a lifelong role to care for their child, unlike Montgomery’s final phase, where the caregiver experiences relief when the care recipient moves to a different setting such as a nursing home [50]. Similar caregiver models for children with disabilities and elderly people [52], or specifically caring for a child with a rare disease [53] have been proposed without consensus so far. Our proposed model contributes to the discussion by emphasizing the dynamic emotional processes linked to the evolving and unpredictable nature of a child’s condition over time. Unlike Gómez-Zúñiga and colleagues [53], this model does not delineate distinct pathways that parents follow in reconceptualizing their identity; instead, our model highlights the turbulent process of identity development for caregivers of children with undiagnosed and rare conditions.

### Implications for psychological counselling and health care services

The present findings highlight the importance of psychological support and interventions for parents. This was also highlighted by recent report from Genetic Alliance UK which found that parents are often having to 'battle' services and fill in information gaps between care providers which is extremely stressful and damages physical and mental health [54].

In a time-constrained clinical setting, screening tools may help identify parents in need of emotional support [55]. However, these tools often fail to capture the dynamic and evolving nature of mental health in parents caring for a child with a rare and undiagnosed condition. Therefore, if used we recommend the regular application of these measures, rather than as a one-off assessment. As demonstrated in this study, the pressure to appear ‘heroic’ or resilient can contribute to emotional needs being overlooked or underreported. Accordingly, long-term psychological guidance in multiple sessions from the moment parents embark the ‘diagnostic odyssey’ may be required, and not only at the time of diagnosis (which many parents may not receive) as highlighted in previous research [56]. Our recommendations 1, 2, and 6 align with recent suggestions from Rareminds UK, specifically that families need access to support at various time points during *“the rare disease journey to help address family dynamics and personal life choices”*. Additionally, Rareminds advocates the need for support to help couples and families manage the impact of rare conditions on personal relationships [57].

Genetic Alliance UK has proposed practical steps for Recommendations 3 and 4, such as the establishment of unified communication pathways like “Team Around the Child” meetings and agreed-upon care plans to potentially mitigate financial barriers [58]. The UK Government has committed to investing £2.3 billion in additional funding to expand and enhance mental health services across England, aiming to provide essential mental health support to an additional 2 million people, including those with rare conditions [59].

The UK Government is actively pursuing recommendation 6 including through mental health awareness training for healthcare professionals, and workforce education initiatives such as webinars and resources developed with patient input available on the National Genomics Education platform. Additionally, NHS England’s service specifications for patients with rare diseases now mandate the consideration of users’ psychosocial needs as well as the implementation of coordinated pathways for accessing mental health support, as outlined in the “England Rare Diseases Action Plan 2024” [60].

Lastly, our findings may contribute to the discussion of defining and understanding the term “family caregiver”, relating to a recent discussion led by Care Alliance Ireland, who acknowledged the variability of the term across sectors, with its meaning and impact varying by context [11]. 

### Strength and limitations

A key strength of this research is that thematic power was reached in terms of adequate depth of understanding to meet the study aims, in line with recommendations by Braun and Clarke [33]. Another strength is that we were able to involve a particularly vulnerable group of parents who are struggling with their mental health, often overlooked in the rare disease literature, and give them the opportunity to share their experiences and have their story heard.

We purposively sampled parents with medium to high anxiety as well as parents who had lower health-related quality of life and family functioning and/or lower resilience. Our findings therefore need to be interpreted as reflecting a very particular group of parents at a particular timepoint (undergoing WGS). Given that most children in the study had developmental delay, our findings may not be generalisable to parents of children with other rare conditions. Additionally, since most parents reported that their child’s condition had a significant impact on them, the results may not be applicable to parents of less severely affected children. For instance, results from a quantitative meta-analysis demonstrated that parents of children affected by autism spectrum disorders experience significantly more stress than parents of children with other disabilities [61].

The majority of participants in this study were white British, highlighting the need for further research involving ethnic minority groups to determine if the findings are generalizable. In our sample, 32.1% (*n* = 17) of the 53 white British individuals invited to participate agreed to be interviewed. Among the 11 Asian or Asian British individuals invited, 45.5% (*n* = 5) agreed to participate, while none of the 4 Black or Black British individuals contacted chose to participate, highlighting the importance of further work with this group in particular. Shea et al. [62] found that amongst African Americans, factors such as limited awareness of research participation opportunities and mistrust of clinical research played a major role in why they were underrepresented in research.

Furthermore, only four fathers agreed to participate, perhaps reflecting known gender inequalities in family caregiving as also reported in this study. Based on our sampling criteria for poor mental health, fathers tended to score better on the scales employed in this study. Consequently, only a limited number of fathers were invited to participate, nonetheless we also found that these fathers were less responsive to our interview invites. This outcome may relate to less interest more generally in taking part in research, measures that are less sensitive for men, or the lower emotional burden experienced by fathers, as suggested by our findings. This aligns with a report which showed that fathers exhibit lower levels of emotional distress compared to mothers, particularly in the context of depression and anxiety [3]. It is not possible to make general statements about caregiving and fatherhood based on such a small sample.

## Conclusion

This study highlights the complexity associated with emotional wellbeing and identity amongst parents of children with undiagnosed and rare conditions, who may be coping less well. This particular group of parents may benefit from psychological support at various time points, warranting further research on when and how this might most effectively be delivered. Additionally, it raises the question of whether returning WGS results and improving current health-care services might alleviate (to some degree), the burden of uncertainty, thereby increasing parents’ emotional wellbeing. This is an area for further exploration.

## Electronic Supplementary Material

Below is the link to the electronic supplementary material.

Supplementary Material 1

## Data Availability

The qualitative data from this study, consisting of anonymized interview transcripts, are not publicly available due to the sensitive nature of the topics discussed and participant consent agreements. However, excerpts of transcripts relevant to the study findings can be shared upon reasonable request to the corresponding author.

## Abbreviations

BRS: Brief Resilience Scale
GAD-7: General Anxiety Disorder 7
GMS: Genomic Medicine Service
NHS: National Health Service
PPI: Patient and public involvement
PED-QL: Pediatric Quality of Life Questionnaire
(W)GS: (whole) genome sequencing
